# Determinants of 3Rs behaviour in plastic usage: A study among Malaysians

**DOI:** 10.1016/j.heliyon.2020.e05805

**Published:** 2020-12-23

**Authors:** Loh Chun T'ing, Krishna Moorthy, Chin Yoon Mei, Foo Pik Yin, Wong Zhi Ying, Chin Wei Khong, Gan Zhao Chern, Thong Zin Lin

**Affiliations:** a Faculty of Business and Finance, Universiti Tunku Abdul Rahman, Kampar Campus, Perak, Malaysia; b School of Economics and Management, Xiamen University Malaysia, 43900, Sepang, Selangor, Malaysia

**Keywords:** Plastic waste, Theory of planned behaviour (TPB), Habit, Facilitating conditions, Reduce, reuse, and recycle (3Rs), Malaysia

## Abstract

This research was conducted to explore the factors affecting Malaysians’ application of reduce, reuse and recycle (3Rs) concept in plastic usage. This study adopted variables from the Theory of Planned Behaviour (TPB), namely, attitude, subjective norm and perceived behavioural control and added on two more variables, habit and facilitating conditions to study the plastic usage. Self-administered questionnaires were used to collect the data and analysis done. The results showed that all variables influence the plastic usage behaviour. This research contributes to a better understanding of the relationship between the determinants of behavioural intention of 3Rs application on plastic usage. Through the suggestions of suitable strategies, this research would contribute to reducing environment pollution caused by plastic waste.

## 1 Introduction

The excessive production and consumption of plastic have caused plastic pollution. Plastic pollution is a global concern as it brings profound impact to the society and the environment. As plastic composes of non-biodegradable substance, there is a lack of safe ways to dispose of plastic waste. The poisonous chemical exposes human, animals and environment to harm (Koushal et al., 2014).

Among the attempts to control plastic crisis, Denmark was the first country that imposed levy on manufacture of plastic bag; countries such as Albania, Bangladesh, China, Israel banned single-use plastic shopping bags; United Kingdom imposed 0.05 pounds sterling for a plastic bag and two largest supermarket chains refused to provide free plastic bags in Australia (Klein, 2018).

### 1.1 Problem statement

According to Geyer et al. (2017), 8300 metric tons of plastic were produced from the year 1950–2017 in the worldwide, which 6400 metric tons were used and wasted; 79% in landfills; 9% were recycled and the remaining 12% were being incinerated. Malaysia was ranked the eighth in the world's top ten list on mishandled plastic waste (Ministry of Energy, Science, Technology, Environment & Climate Change, 2017). According to Malaysia Housing and Local Government Minister, population of Malaysia was 32 million and every day there are 38,000 metric tons of waste being generated (Lim, 2018). From the waste generated, 47% was contributed by food waste and other organic waste followed by paper 15%, plastics 14%, and others 24%. Only 24% of waste were recycled and the remaining 76% were landfilled (Jereme et al., 2015).

For sustainable development, Malaysia government had developed a “Roadmap towards Zero Single-use Plastic”. The Government intended to phase out the disposable plastic like straw and replace plastic bag with bio bag by 2030. In year 2011, Malaysia Federal Government had launched No-Plastic Bag Day (NPBD) campaign every Saturday. Levy will be imposed if plastic was requested by consumer; but only selected places such as hypermarkets, retail outlets, and supermarkets follow the campaign. The purpose of this campaign is to reduce plastic usage and negative impact towards the environment. The effectiveness of the program was a 52.3% reduction and remaining 47.7% chose to pay for the levy (Asmuni et al., 2015).

In year 2014, The Malaysian Plastics Manufacturers Association (MPMA) also promoted reduce, reuse and recycle (3Rs) programme to raise public awareness. According to Ahmadi (2017), Malaysia's household participants in 3R projects were ranged between medium low to medium, and the study revealed that only 26% of the households take part in waste reduction activities and 20% in practice reuse and 29% in separation at source.

### 1.2 Deficiencies of past studies

Many past studies selected University students or young population as their target respondents and, therefore, the research samples were skewed to the young population. They suggested that a wider range of target respondents should be investigated as young and senior respondents might have a different perspective (Hasan et al., 2015; Tiew et al., 2013; Sun et al., 2017; and Lin et al., 2017).

The researches of Lin et al. (2017), Sun et al. (2017) and Ali and Yusof (2018) focused on the determinants of the intention behaviour. They suggested that future studies should investigate the actual behaviour of respondents as there was a gap between intention and actual behaviour. Past studies that were adopted Theory of Planned Behaviour (TPB) to examine users’ behaviour on reducing, reusing, and recycling of plastic waste were common. Some researchers suggested that future researches should include habits and facilitating conditions, as these factors will affect the recycling behaviour (Tonglet et al., 2004; Xu et al., 2017).

The past researches on environmental behaviour in Malaysia are scarce and the researches done in other developing countries may not be able to be generalised to Malaysia. Therefore, this research would be useful for future researchers in pursuing research on pro-environmental behaviour in Malaysia.

### 1.3 Research objectives

This research aims to investigate the factors that will affect Malaysians’ application of the 3Rs concept in plastic usage. The general research objective has been narrowed down to the following specific research objectives:

1. To investigate the relationship between attitude and Malaysians' 3Rs behavioural intention in plastic usage.
2. To investigate the relationship between subjective norm and Malaysians' 3Rs behavioural intention in plastic usage.
3. To investigate the relationship between perceived behavioural control and Malaysians' 3Rs behavioural intention in plastic usage.
4. To investigate the relationship between Malaysians' 3Rs behavioural intention in plastic usage and their 3Rs behaviour.
5. To investigate the relationship between habit and Malaysians' 3Rs behaviour in plastic usage
6. To investigate the relationship between facilitating conditions and Malaysians' 3Rs behaviour in plastic usage.

### 1.4 Practical/social contribution

This research aims to stimulate Malaysians’ environmental motives that turn their intentions into actual 3Rs behaviour. This research will help to reduce environmental pollution caused by plastic waste through the recommendation of suitable strategies to aid the government and corporations which can influence the society to apply the 3Rs in plastic usage.

### 1.5 Theoretical contribution

This research has adapted TPB and supplemented it with two variables, which are habit and facilitating conditions. It offers future researchers a research model towards the 3Rs concept in plastic usage. This can facilitate better understanding of the relationship between behavioural intention and actual behaviour of applying of 3Rs concept on plastic usage.

## 2 Literature review

### 2.1 Theory of Planned Behaviour (TPB)

TPB was advocated by Icek Ajzen in the year 1991 and it extends from Theory of Reasoned Action (TRA) by Fishbein and Ajzen (1975). The deviation between TPB and TRA is that TPB included perceived behavioural control as a new variable (Robinson 2010). Thus, TPB is formed to have three socio-psychological and behaviour-specific factors, which are attitude, subjective norm and perceived behavioural control to predict the intention and intention become the actual behaviour (Heinrich, 2016).

TPB is a significant and frequently cited model to predict individual's social behaviour (Ajzen, 2011). Several studies had used TPB to examine the pro-environmental behaviour. TPB was used to measure the behaviour of University students in reducing plastic consumption (Hasan et al., 2015) and to explain the recycling behaviour of household (Strydom, 2018). Thus, TPB is a suitable model to examine the factors affecting reduce and recycling behaviour. However, according to Wayne (2018), although TPB was used for much research on the pro-environmental behaviour, it has limitations in predicting the behaviour that is routine or repeated. Jennifer, Kathleen, Paul and Laura (2014) concurred with this TPB's limitation. Moreover, it also assumed that opportunity or resources are provided to perform the action. Tonglet et al. (2004) posited that recycling is an action that is repeated and performed automatically without any need to consciously remember. Therefore, habit, a variable from the Theory of Interpersonal Behaviour (TIB), has been included to predict the recycling behaviour. Furthermore, in order to perform recycling behaviour, it requires facilitating conditions such as time, space and availability of facilities. Without resources, it will restrain individual to perform the recycling behaviour. Thus, another variable from TIB, facilitating conditions, was also added to this research.

### 2.2 Theory of Interpersonal Behaviour (TIB)

The Theory of Interpersonal Behaviour (TIB) was advocated by Triandis (1977). TIB belongs to a school of cognitive models, namely that of Fishbein and Ajzen's (1975) Theory of Reasoned Action and Ajzen’s (1991) Theory of Planned Behaviour (Milhausen et al., 2006). TIB model added habits and facilitating conditions that would impact the behaviour (Robinson 2010). Most of the studies included facilitating conditions or habit to examine waste management, waste separation or recycling behaviour (Aljerf, 2018). Some studies have shown that habit and facilitating conditions have a significant impact on the waste recycling behaviour. They had been used in the study of Khalil et al. (2017); Teo (2016); Ittiravivongs (2012); and Davis et al. (2006). The results showed that habit and facilitating conditions had an impact on the recycling behaviour. In the TIB model, facilitating conditions is a moderating variable. However, in the study of Latif et al. (2018), facilitating conditions was found to have significant impact on actual recycling behaviour as an independent variable. Hence, in this research, facilitating conditions is examined as an independent variable of the 3Rs behaviour.

### 2.3 3Rs behaviour

This research examines behaviour by applying the 3Rs concept - reduce, reuse and recycle, which is a basic practice for managing waste. Reduce indicates avoidance of usage. Some States in Malaysia had conducted a campaign to educate, raise awareness and encourage public to consume product that produce less waste. For example, use of bio-bag or recycling bag to replace single-use plastic bag and steel straw to replace plastic straw (Ahmadi, 2017).

Reuse can be performed through selling, donating or repairing items; therefore, it can reduce waste. For example, use beverage bottle to refill water rather than purchase a new bottle; use reusable cups instead of disposable cups; and reuse plastic bags for trash collection (Ahmadi, 2017).

Recycling includes collection, separation and processing of waste. Paper, plastic, metal and glass are recyclable materials (Ahmadi, 2017). Research showed that the 3Rs behaviour has a positive impact on solving environmental problems (Nindyati, 2014).

### 2.4 Attitude

Attitude is a set of feelings, beliefs, and behaviours toward an event or a situation (Chaiklin, 2011). Attitude has a significant positive relationship with behavioural intention to reduce usage of plastic, nevertheless the relationship was infirm (Hasan et al., 2015). Lin et al. (2017) found that a positive relationship exists between attitude and behavioural intention on both mandatory and voluntary pro-environmental programme. However, the score of attitude to the behavioural intention of the mandatory program was lower than voluntary program. Furthermore, Chang and Chou (2018) concluded that attitude has positive and direct influence on customers’ behavioural intention to “Bring Your Own Shopping Bags” (BYOB) with grocery shopping in Taiwan. The findings of Wan et al. (2012) indicated that behavioural intention with regard to recycling is influenced by the attitude. This is concurred by Pamuk and Kahriman-Pamuk (2019) that attitude is one of the significant predictors of recycling intentions. Thus, a hypothesis was developed:

H1 There is a significant and positive relationship between attitude and 3Rs behavioural intention.

### 2.5 Subjective norm

Subjective norm is defined as a social pressure from others who are essential towards an individual to behave in a certain manner (Finlay et al., 1999). Goh, David and Ahmed (2018) found that subjective norm has a positive and significant relationship towards households recycling behavioural intention and concluded that support from families, friends and the society will affect the level of recycling behavioural intention. In addition, in the research of Lee and Tanusia (2016), subjective norm was found to have significant and positive relationship towards energy conservation intention. The findings indicated that family, friends including the community can influence and encourage students in energy conservation. Based on the discussion above, a hypothesis is developed as below:

H2 There is a significant and positive relationship between subjective norm and 3Rs behavioural intention.

### 2.6 Perceived behavioural control

Perceived behavioural control is a person's confidence that can regulate self-internal condition and action, stimulate and react to the given situation, and achieve the desired outcome (Wallston et al. 1987). Mamun, Mohiuddin, Ahmad, Thurasamy, and Fazal (2018) studied the determinants of recycle intention and behaviour among low salary family. The findings revealed that there is a positive relationship between perceived behavioural control and intention on recycling behaviour. Latip, Sharkawi, Sharifuddin, and Mohamed (2018) investigated the impact of attitude, subjective norm and perceived behavioural control toward the intention to comply the practices of environmental management and concluded that intense perceived behavioural control will have higher intention to comply practices of environmental management.

Thus, the following hypothesis is developed:

H3 There is a significant and positive relationship between perceived behavioural control and 3Rs behaviour intention.

### 2.7 3Rs behavioural intention

Behavioural intention is known as the motivational factor that affect one's willingness to execute an action (Ajzen, 2012). Strong intention can be reflected by students' confidence to demonstrate their determination in decreasing plastic usage (Hasan et al., 2015). Strydom (2018) examined the behavioural intention related to household recycling in South Africa. The result revealed that behavioural intention to recycle holds a significant and positive result on behaviour of recycling. Moreover, consumers' behavioural intention to use fabric bag was found to positively affect the behaviour to use lesser disposable bags (Ari and Yilmaz, 2016). Thus, it is hypothesised that:

H4 There is a significant and positive relationship between 3Rs behavioural intention and 3rs behaviour.

### 2.8 Habit

Habit is a comparatively stable behavioural pattern that has been reinforced in the past and can be carried out without cautious deliberation, and is the outcome of automated processes, contrasting to controlled processes like knowingly made decisions (Verplanken and Holland, 2002). According to Wang et al. (2018), habits or past behaviours are essential in shaping behaviours that are routine. Once residents have electricity-saving habits, they will automatically and continuously perform the behaviour. Besides, the findings in Russell et al. (2017) showed that past behaviour was influential in consumers’ intentions and actual behaviour on decrease food waste. Thus, it is hypothesised that:

H5 There is a significant and positive relationship between habit and 3Rs behaviour.

### 2.9 Facilitating conditions

Facilitating conditions is a situational factor or opportunity that encourage or discourage a person towards a specific behaviour (Venkatesh et al., 2012). According to Tiew et al. (2013), there was a positive reaction of students towards recycling movement in the Universiti Kebangsaan Malaysia campus, if the facilities are easy to access. Besides, easy access to recycling facilities would reduce the behavioural costs and motivate society to follow the trend (Zhang et al., 2016). Thus, it is hypothesised that:

H6 There is a significant and positive relationship between facilitating conditions and 3Rs behaviour.

**Figure 1:**
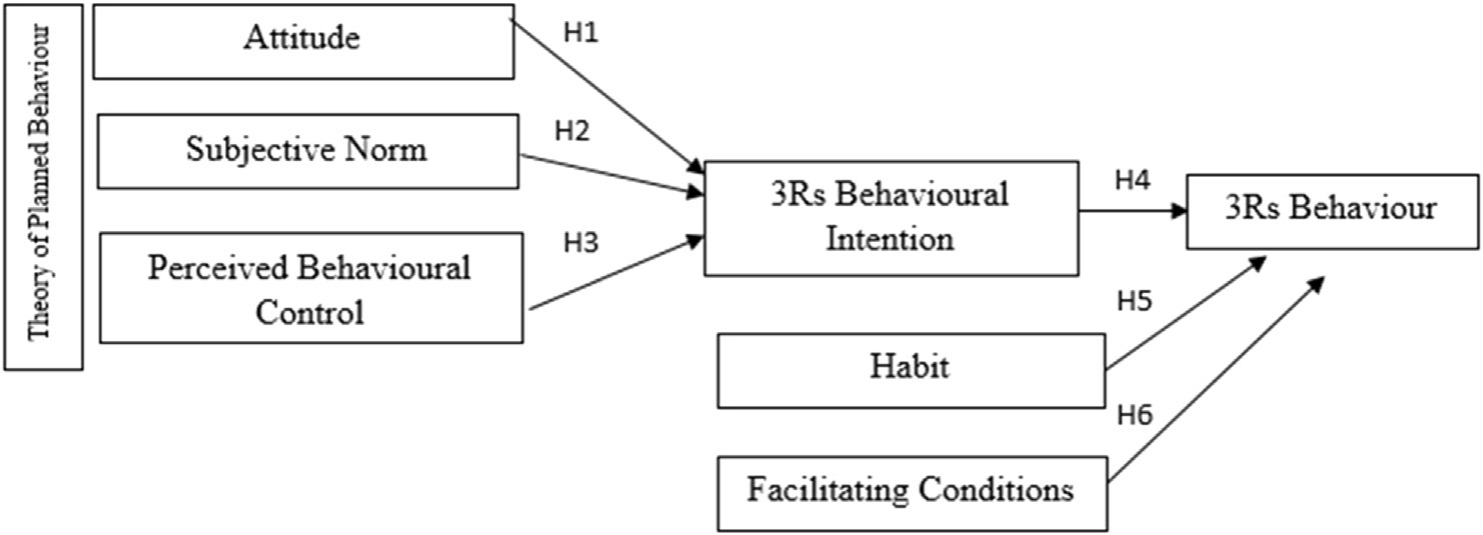
Research model.

Adapted from Ajzen's Theory of Planned Behaviour (1991) and Triandis' Theory of Interpersonal Behaviour (1977)

The research model is thus developed as in Figure 1 below:

## 3 Research methodology

Cross-sectional method and self-administered survey approach was used in this research. Interval five-point Likert scale method was chosen for the questionnaires, as it is a reliable and responsive measurement (Vickers, 1999).

The target respondents in this research are Malaysians aged 18 years old and above because they have the fundamental consuming ability and logical thinking. As stated by Malaysia Age of Majority Act 1971, males and female in Malaysia at the age of eighteen should be considered as majority (Lawyerment, 2020).

Malaysia is a federation country which consists of 13 States and 3 Federal States. Based on the demographic statistics of Malaysia in the first quarter of 2019, the top five population States in Malaysia are Selangor, Sabah, Johor, Sarawak, and Perak in the sequence of population (Department of Statistics Malaysia, 2019). As Sabah and Sarawak are located in East Malaysia, samples were not selected due to time, geographical and cost constraint. The sampling data were thus, collected from Selangor, Johor and Perak which had 39.3% of the population in Malaysia. As the list of the complete population is not available in this research, there was no sampling frame for this research. Thus, non-probability sampling technique was adopted in this research using quota sampling.

The appropriate sample size based on the item-to-response-ratio should be 1:4 to 1:10 (Hinkin, 1998). There were 34 items in this research, the sample size should range from 136 to 340. The result is more likely to be significant if the sample size is more than 10 and less than 2,000; the characteristic of sampling distribution will also be affected if the sample size is too small (Field, 2013). Hence, 400 sets of survey were considered sufficient to represent the population.

### 3.1 Pre-test and pilot test

After completed designing the draft questionnaire, but before the actual survey was conducted, a pre-test was carried out where the questionnaire questions were inspected and verified by 3 experts in the related field. Adjustments were made according to advices and recommendations of the experts.

After that, a pilot test was conducted for 30 samples to test the validity of items in the questionnaire (Appendix). According to Johanson and Brooks (2010), the sample size for pilot test is reasonable within the range of 30–50. Thus, 30 sets of questionnaire were distributed in Kampar, Perak State on 3 June 2019 to perform reliability and normality tests.

Cronbach's alpha was used to measure the internal consistency of questionnaire or test items (Bonett and Wright, 2014). The pilot test results showed that Cronbach's alpha for all variables ranged from 0.728 to 0.906. The acceptable benchmark for Cronbach's alpha value is more than 0.70 (Cortina, 1993). Since all results exceeded the benchmark of 0.7, it was concluded that all items had good internal consistency and were highly reliable.

Skewness and kurtosis tests were used to perform the normality test in this research as skewness and kurtosis are commonly used to picture the normal distribution of independent variables and dependent variable in the form of shape and characteristics (Joanes and Gill, 1998). In the pilot test, the highest value was 0.725 for skewness and 2.027 for kurtosis, whereas the lowest value of was -1.181 and -1.460 respectively. The skewness and kurtosis are considered as normally distributed if the values fall within ±3 and ±10 respectively (Kline, 2005). As the skewness and kurtosis values for all items were within the benchmark, the normality requirement had been achieved.

## 4 Data analysis

### 4.1 Descriptive analysis

The data collection was made from 6 to 15 June 2019 and the locations were hypermarkets and shopping malls within the sampling locations in Selangor, Johor and Perak states. 400 sets of survey questionnaires were disseminated while 339 sets of useable questionnaires were collected, indicating a feedback rate of 84.75%.

Table 1 shows that there were 178 female respondents (52.5%) and 161 male respondents (47.5%). Among the 339 respondents, 162 (47.8%) respondents were from Selangor, 96 (28.3%) from Johor and the remaining 81 (23.9%) from Perak. There were 170 (50.1%) Malay, 105 (31%) Chinese, 56 (16.5%) Indian and the remaining 8 (2.4%) from other races. The results show that most of the respondents are Malay. Among the 339 respondents, 142 (41.9%) were aged between 18 to 30, followed by 113 (33.3%) respondents within the age group of 31–40, while 84 (24.8%) aged 41 years and above. Among the 339 respondents, 78 (23%) completed high school; 96 (28.3) were diploma holders; 125 (36.9%) were degree holders and the remaining 40 (11.8%) were in other categories. The result reveals that, degree holders were the largest portion of respondents. As for monthly income, there were 94 (27.7%) respondents with a monthly income of less than RM1,000, 84 (24.8%) between RM1,001 to RM2,000, 95 (28%) between RM2,001 to RM3,000 and 66 (19.5%) more than RM3,000.

**Table 1:** Demographic profile of the respondents.

	Frequency	Percentage (%)
Gender		
- Male	161	47.5
- Female	178	52.5
Residential Areas of Respondents		
- Selangor	162	47.8
- Johor	96	28.3
- Perak	81	23.9
Race		
- Malay	170	50.1
- Chinese	105	31.0
- Indian	56	16.5
- Other	8	2.4
Age		
- 18-30	142	41.9
- 31-40	113	33.3
- >41	84	24.8
Highest education level		
- High school	78	23.0
- Diploma	96	28.3
- Degree	125	36.9
- Others	40	11.8
Monthly income		
- < RM1,000	94	27.7
- RM1,001-RM2,000	84	24.8
- RM2,001-RM3,000	95	28
- > RM3,000	66	19.5

### 4.2 Central tendencies measurement of constructs

Mean and standard deviation of all the items in this research are presented in Table 2. A5 has the highest mean value at 4.445 whereas SN2 has the lowest mean value of 3.909. Standard deviation for all variables falls between 0.6269 (A2) to 0.8828 (PBC 6).

**Table 2:** Statistics of constructs’ central tendencies measurement.

	Items	N	Mean	Standard Deviation
*Dependent Variable*				
3Rs Behaviour (B)	B1	339	4.292	0.7380
	B2		4.097	0.8209
	B3		4.212	0.7023
	B4		4.106	0.7579
	B5		4.227	0.6998
	B6		3.953	0.8758

*Independent Variables*				
Attitude (AT)	A1	339	4.307	0.6438
	A2		4.283	0.6269
	A3		4.295	0.6538
	A4		4.254	0.7422
	A5		4.445	0.6875
Subjective Norm (SN)	S1	339	3.965	0.8241
	S2		3.909	0.7810
	S3		3.938	0..8025
	S4		4.041	0.8521
Perceived Behavioural Control (PBC)	PBC1	339	3.973	0.7971
	PBC2		4.183	0.6542
	PBC3		4.165	0.7272
	PBC4		4.274	0.7123
	PBC5		4.192	0.7547
	PBC6		4.041	0.8828
3Rs Behavioural Intention (BI)	BI1	339	4.156	0.7312
	BI2		4.147	0.7510
	BI3		4.183	0.7628
	BI4		4.156	0.7472
	BI5		4.233	0.7470
Habit (HB)	H1	339	4.041	0.7245
	H2		3.997	0.7154
	H3		4.009	0.7438
	H4		4.053	0.7902
Facilitating Conditions (FC)	F1	339	4.242	0.6710
	F2		4.091	0.7579
	F3		4.304	0.7002

It can be seen that the Mean value of dependent variable, 3Rs behaviour, ranged from 3.953 to 4.292, whereas the mean values of 3Rs behavioural intention ranged from 4.147 to 4.233; attitude ranged from 4.254 to 4.445; subjective norm ranged from 3.909 to 4.041; perceived behavioural control ranged from 3.973 to 4.274; habit ranged from 3.997 to 4.053 and facilitating conditions ranged 4.091 to 4.304. Standard deviation for all variables were between 0.6269 (A2) to 0.8828 (PBC 6).

### 4.3 Scale measurement

Table 3 shows that all variables had a Cronbach's alpha score of above the benchmark level of 0.7. Facilitating conditions had the lowest value of 0.809 while subjective norm had the highest value of 0.870. Hence, all items had good internal consistency.

**Table 3:** Summary of the reliability test.

Variables	Cronbach's Alpha Values	Number of Questionnaire Items
3Rs Behavioural Intention	0.851	5
Attitude	0.840	5
Subjective Norm	0.870	4
Perceived Behavioural Control	0.824	5
Habit	0.858	4
Facilitating Conditions	0.809	4
3Rs Behaviour	0.820	6

### 4.4 Normality test

Table 4 shows that the skewness values range from -0.361 to -1.286 and the kurtosis values range from -0.225 to 2.605. The item achieved the highest value of skewness was HB3 (-0.361) and the highest value of kurtosis was AT5 (2.605); whereas the lowest value of skewness was AT 5 (-1.286) and the lowest value of kurtosis was HB3 (-0.225). As all the items had skewness values within the benchmark of ±3 and kurtosis values within the benchmark of ±10, the normality requirement had been achieved.

**Table 4:** Summary of normality test.

Variables	Items	Skewness	Kurtosis
Attitude (AT)	AT1	-0.722	-1.414
	AT2	-0.731	2.015
	AT3	-0.900	2.176
	AT4	-0.926	1.378
	AT5	-1.286	2.605
Subjective Norm (SN)	SN1	-0.572	0.401
	SN2	-0.438	0.316
	SN3	-0.544	0.360
	SN4	-0.858	1.069
Perceived Behavioural Control (PCB)	PBC1	-0.552	0.197
	PBC2	-0.653	1.569
	PBC3	-0.682	0.986
	PBC4	-0.751	0.643
	PBC5	-0.790	0.738
	PBC6	-0.885	0.757
3Rs Behavioural Intention (BI)	BI1	-0.845	1.564
	BI2	-0.797	1.176
	BI3	-1.126	2.551
	BI4	-0.691	0.583
	BI5	-0.836	1.053
Habit (HB)	HB1	-0.391	-0.093
	HB2	-0.386	0.059
	HB3	-0.361	-0.225
	HB4	-0.710	0.723
Facilitating Conditions (FC)	FC1	-0.682	1.140
	FC2	-0.810	1.487
	FC3	-0.811	0.582
	FC4	-1.093	1.587
3Rs Behaviour (B)	B1	-1.146	2.182
	B2	-1.053	1.869
	B3	-0.992	2.399
	B4	-1.041	2.425
	B5	-0.814	1.646
	B6	-0.626	0.177

### 4.5 Structural equation modeling

To confirm the reliability and validity of the data and the model fit, a structural equation modelling analysis has been performed through SmartPLS. The composite reliability for all constructs were found to be in the range of 0.899 and 0.952, exceeding the threshold of 0.7, indicating that these constructs have high levels of internal consistency reliability (Hair et al., 2017). The constructs also portrayed high levels of convergent validity as the Average Variance Extracted (AVE) were found to range between 0.600 and 0.832, which exceeded the cut-off value of 0.5. Moreover, the discriminant validity of the constructs was assessed using Fornell-Larcker criterion. All the diagonal values were found to be greater than the off diagonal inter-construct correlation values, suggesting that there is discriminant validity. Regarding model fit, a SRMR of 0.086 has been achieved. SRMR indicates the acceptable fit when it produces a value smaller than 0.10 (Kline, 2011).

### 4.6 Inferential analysis

Table 5 presents the summary of Pearson Correlation, which is a simplified one-sided matrix. According to the table, the relationship between the variables are positive and significant. The strongest relationship to BI was found to be PBC (0.643), followed by AT (0.627), SN (0.562), B (0.544), Habit (0.307) and the weakest relationship was found to be FC (0.0114). The strongest relationship to B was found to be PBC (0.684), followed by HB (0.602), BI (0.544), SN (0.482), AT (0.415) and lastly FC (0.413).

**Table 5:** The summary of Pearson Correlation.

Pearson's Correlation Matrix	BI	AT	SN	PBC	HB	FC	B
BI	1.000						
AT	0.627 <0.001	1.000					
SN	0.562 <0.001	0.400 <0.001	1.000				
PBC	0.643 <0.001	0.496 <0.001	0.579 <0.001	1.000			
HB	0.307 <0.001	0.422 <0.001	0.556 <0.001	0.658 <0.001	1.000		
FC	0.114 <0.001	0.416 <0.001	0.356 <0.001	0.456 <0.001	0.446 <0.001	1.000	
B	0.544 <0.001	0.415 <0.001	0.482 <0.001	0.684 <0.001	0.602 <0.001	0.413 <0.001	1.000

Overall, the strongest relationship existed between PBC and B at 0.684; whereas the weakest relationship existed between BI and FC at 0.114. The Sig (2-tailed) value for all variables did not exceed 0.05, demonstrating a correlation between all variables were statistically significant. As the result showed that the correlation between all the variables ranged within 0.114 and 0.658, no multicollinearity problem existed, since the correlation values were less than 0.90 (Wissmann et al., 2007).

### 4.7 Multiple linear regression (MLR) analysis

There are two MLR equations in this research. The first MLR equation examined the relationship between attitude, subjective norm, perceived behavioural control and 3Rs behavioural intention, while the second MLR equation examined the relationship between 3Rs behavioural intention, habit, facilitating conditions and 3Rs behaviour.

**The First MLR Equation**

(1) 3Rs BI = β0 + β1AT+ β2SN + β3PBC

BI, Behaviour Intention

β0, Constant term

β, Scope of Coefficient

AT, Attitude

SN, Subjective Norm

PBC, Perceived Behavioural Control

Table 6 shows that the R value was 0.756 which indicated a high level of correlation, while the R^2^ value was 0.571 which indicated that 57.1% of the 3Rs BI can be explained by the variables tested in this study. The remaining 42.9% can be expounded by other predictors which did not tested in this study.

**Table 6:** The first MLR equation model summary.

Model	R	R^2^	Adjusted R^2^	Std. Error of the Estimate
1	0.756	0.571	0.567	0.38955

Table 7 shows that the F-value was 148.659 and p-value was 0.000. Since F-value was large and p-value did not exceed 0.05, it signifies that at least one independent variable can be used in modelling the dependent variable. Hence, model fit was achieved in this research.

**Table 7:** Summary of ANOVA

Model		Sum of Squares	df	Mean Square	F	Sig.
1	Regression	67.676	3	22.559	148.659	0.000
	Residual	50.836	335	0.152		
	Total	118.512	338			

Based on the coefficients from Table 8, the first MLR equation is formulated as below:

BI = 0.140 + 0.423 (AT) + 0.190 (SN) + 0.352 (PBC)

**Table 8:** Summary of coefficients.

Model		Unstandardized Coefficients		Standardized Coefficients		
		B	Std. Error	Beta	t	Sig.
1	(Constant)	0.140	0.195		0.718	0.474
	AT	0.423	0.047	0.375	8.975	0.000
	SN	0.190	0.038	0.222	4.990	0.000
	PBC	0.352	0.050	0.329	7.009	0.000

Table 8 shows that the p-values for attitude, perceived behavioural control and subjective norm were all below 0.05. This indicates that there are significant positive relationships between all three independent variables: attitude, subjective norm and perceived behavioural control and 3Rs behavioural intention. Thus, Hypotheses 1 to 3 were supported. Among the independent variables, attitude had the highest beta of 0.423 which signifies the strongest positive significant relationship with 3Rs behavioural intention. Perceived behavioural control ranked the second with a beta value of 0.352, followed by subjective norm with a beta value of 0.190.

**The Second MLR Equation**

(2) 3RsB = β0 + β43Rs BI + β5HB + β6FC

B, Behaviour

HB, Habit

FC, Facilitating Conditions

Table 9 shows that the R value was 0.652 which indicated a high degree of correlation, while the R^2^ value was 0.425 which meant that 42.5% of the sum variation in the dependent variable (3Rs behaviour) can be illustrated by the independent variables.

**Table 9:** The second MLR equation model summary.

Model	R	R-square	Adjusted R-square	Std. Error of the Estimate
1	0.652	0.425	0.420	0.42467

Table 10 shows that the F-value was 82.496 and the p-value was 0.000. Since the F-value was large and p-value did not exceed 0.05, it indicated that at least one independent variable can be used in modelling the dependent variable. Hence, model fit was achieved in this research.

**Table 10:** Summary of ANOVA

Model		Sum of Squares	DF	Mean Square	F	Sig.
1	Regression	44.633	3	14.878	82.496	0.000
	Residual	60.415	335	0.180		
	Total	105.048	338			

Based on the coefficients in Table 11, the second MLR equation is formulated as below:

B = 1.244 + 0.234 (BI) + 0.354 (H) + 0.120 (FC)

**Table 11:** Summary of coefficients.

Model		Unstandardized Coefficients		Standardized Coefficients		
		B	Std. Error	Beta	t	Sig.
1	(Constant)	1.244	0.199	-	6.249	0.000
	BI	0.234	0.051	0.248	4.612	0.000
	H	0.354	0.048	0.396	7.359	0.000
	FC	0.120	0.046	0.125	2.620	0.009

Table 11 shows that the p-values for 3Rs behavioural intention, habit and facilitating conditions were all below 0.05. which means that they all have a significant positive relationship with 3Rs behaviour. Thus, Hypotheses 4 to 6 were supported. Habit has the highest beta value of 0.354 and signifies that it has the strongest significant positive relation with 3RsB. It is followed by 3Rs behavioural intention with a beta value of 0.234 and lastly is the facilitating conditions with a beta value of 0.120.

Table 12 below shows the summary of MLR analyses’ results:

**Table 12:** Summary of result.

Hypotheses	Multiple Linear Regression		
	Unstandardized Coefficients Beta	P-Value	Hypothesis
H1: There is a significant positive relationship between 3Rs behavioural intention and 3Rs behaviour.	0.234	0.000	Supported
H2: There is a significant positive relationship between attitude and 3Rs behavioural intention.	0.423	0.000	Supported
H3: There is a significant positive relationship between subjective norm and 3Rs behavioural intention.	0.190	0.000	Supported
H4: There is a significant positive relationship between perceived behavioural control and 3Rs behavioural intention.	0.352	0.000	Supported
H5: There is a significant positive relationship between habit and 3Rs behaviour.	0.354	0.000	Supported
H6: There is a significant positive relationship between facilitating conditions and 3Rs behaviour.	0.120	0.009	Supported

## 5 Discussion

### 5.1 Attitude and 3Rs behavioural intentions

H1 was supported and thus, attitude positively and significantly affects 3Rs behavioural Intention. This is in line with the past studies by Ali and Yusof (2018), Hasan et al. (2015), Lin et al. (2017), Chang and Chou (2018) and Sun et al. (2017). As attitude had the highest coefficient correlation towards behavioural intention with 0.423, it should be given the highest attention. According to the study by Yusof et al. (2019), whilst effort was made by government in encouraging pro-environmental behaviour, there is no significant change in the participation rate of recycling activities. This might be due to lack of awareness, exposure and attitude in the community towards the long-term recycling benefit of recycling. Wan, Cheung, and Shen (2012) suggested that educational and promotional programmes highlighting the benefits and importance of recycling activities could encourage recycling on campus. In this study, the mean for attitude ranged between 4.254 to 4.445, which indicates that respondents agree that they had responsibility towards 3Rs behavioural intention. It can be seen from the attitude questions in the questionnaire (Appendix) that they are more related to the affective component of the attitude. Thus, appropriate project should be carried out to educate Malaysians with positive attitude towards the 3Rs concept on plastic usage. This is with the aim to increase the awareness of the environmental responsibility and to foster a positive attitude towards the environment.

### 5.2 Subjective norm and 3Rs behavioural intention

H2 was supported and thus, subjective norm positively and significantly affects 3Rs behavioural Intention. The result is in line with the studies by Goh et al. (2018); and Lee and Tanusia (2016) but contrary to the study by Ayob and Low (2016), which found subjective norm to be insignificant to the University students’ waste separation intention which indicated that waste separation intention was not influenced by acquaintance, peer and professor. Ali and Yusof (2018) also found that SN does not have a significant relationship with BI. However, as their research was about expatriates to Malaysia and not on Malaysian citizen, the SN influence may not be so substantial.

Although the coefficient correlation of subjective norm towards behavioural intention was the lowest at 0.190 as compared to the attitude and perceived behavioural control, it should still be given sufficient attention. Referring to past studies by Yu et al. (2018), the result of the study showed that the relationship between subjective norm with behavioural intention is weak compared to other variables. It might be due to the culture of the society that does not encourage 3Rs behaviour (Ali and Yusof, 2018). Therefore, people felt less pressure when not performing the 3Rs behaviour. Thus, it is important that the culture of 3Rs concept to be widespread throughout the nation and become a norm of the society. Knowledge and benefits of 3Rs should be included in the syllabus of the education system from a young age to create the awareness and to shape a norm of environment friendly behaviour among the Malaysian citizens regardless of race and cultural differences.

### 5.3 Perceived behavioural control and 3Rs behavioural intentions

Results showed that perceived behavioural control positively and significantly affects 3Rs behavioural Intention and thus, H3 was supported. The result in this study showed that perceived behavioural control had a significant positive relationship with 3Rs behavioural intention. It is consistent with the past studies by Mamun et al. (2018) and Latip et al. (2018) but contradicted to the study by Khan et al. (2019) which found that perceived behavioural control to have an insignificant relationship with recycling intention due to lack of recycling facilities. As the coefficient correlation of perceived behavioural control towards 3Rs behavioural intention was high at 0.352, it should be given the due attention. According to Strydom (2018), perceived behavioural control has a strong influence on recycling behavioural intention as it shows that the community or society feel that they have the ability to apply the 3Rs concept and it is under their control. According to Ling et al. (2018), households of Melaka perceived themselves to have more control over the recycling activities and have strong intention to perform recycle action.

### 5.4 3Rs behavioural intention and 3RS behaviour

The results showed that 3Rs behavioural Intention positively and significantly affects 3Rs behaviour and thus, H4 was supported. According to past studies by Philippsen (2015), Hasan et al. (2015), Ari and Yilmaz (2016) and Strydom (2018), 3Rs behavioural intention have a significant positive relationship with 3Rs behaviour. The 3Rs behavioural intention was affected by attitude, subjective norm and perceived behavioural control and the intention affects the 3Rs behaviour. Using the TPB, the study by Susanto et al. (2019), indicated that the 3Rs behavioural intention has a strong relationship towards the 3Rs behaviour of community in Tanjung Mas, Indonesia. In that research, the correlation between intention and behaviour was found to be high at 0.766. Most of the respondents have intention to apply 3Rs if clear guidance is provided, satisfied with the local 3Rs programme and convinced the advantage of the 3Rs. Thus, it was concluded that waste recycling practice should be enriched with the community in Tanjung Mas.

### 5.5 Habit and 3Rs behaviour

The results showed that habit positively and significantly affects 3Rs behaviour and thus, H5 was supported. According to the past researches by Russell et al. (2017), Wang et al. (2018) and Colesca et al. (2014), habit and 3Rs behaviour have a significant positive relationship. Thus, the result in this study is in line with the past studies. Knussen, Yule, Mackenzie, and Wells (2004) examined the intention to recycle household waste in a geographical area and found that habit has a significant independent influence on recycling intention. However, the findings indicated that past behaviour–intention relationship was stronger for those with no perceived habit of recycling. As habit had the highest coefficient correlation towards 3Rs behaviour at 0.354 which was even higher than 3Rs behavioural intention (0.234), it should be given the highest attention.

According to Mahat et al. (2016), schools, teachers or guardians play an important role in educating children from young with pro-environmental behaviour. Through continuous education of 3Rs concept, it could raise the awareness of children and the application of 3Rs behaviour will become habitual. Environmentally friendly cultural practices such as the use banana leaf to wrap food and use hand to consume food should be encouraged as it can reduce to use of plastic container and single use cutlery. According to Vassanadumrongdee and Kittipongvises (2018), past behaviour has potential to influence future behaviour, habit formation and semiautomatic actions in complex behaviours.

### 5.6 Facilitating conditions and 3Rs behaviour

The results showed that facilitating conditions positively and significantly affects 3Rs behaviour and thus, H6 was supported. According to the past studies by Tiew et al. (2013), Ali and Yusof (2018), and, Harsono and Suryana (2014), facilitating conditions have a significant positive relationship with 3Rs Behaviour. Thus, the result in this study is consistent with the past studies. The coefficient correlation of facilitating conditions towards 3Rs behaviour is the lowest (0.120) compared to 3Rs behavioural intention and habit. However, it should still be given sufficient attention, as it was concluded to have a significant positive relationship with 3Rs behaviour. According to Khalil et al. (2017), lack of recycling facilities affects the respondents' recycling behaviour. Household with better access to recycling facilities and good recycling collection station will improve respondents’ recycling behaviour. In this study, the mean for item FC 3 was the highest among the facilitating conditions. It indicates that the respondents will apply the 3Rs concept if the facilities are provided. Wan, Cheung, and Shen (2012) suggested that educational and promotional programmes highlighting the convenience of the recycling facilities act as the key strategies to encourage recycling on campus.

### 5.7 Theoretical implications

This study concluded that there are positive relationships among the independent variables from TPB, namely, attitude, subjective norm and perceived behavioural control with 3Rs behavioural intention and the independent variables of the 3Rs behavioural intention, habit and facilitating conditions with 3Rs behaviour on plastic usage. The results of this study are in line with the past empirical studies. Research on 3Rs behaviour that associated TPB with habit and facilitating conditions to examine their relationship with 3Rs behaviour are lacking in Malaysia. Hence, this study can add insight to future researchers pursuing research on the issue of pro-environmental behaviour.

Most of the past studies applied TPB to examine the behaviour on reducing and reusing plastic waste; however, this study associated TPB with habit and facilitating conditions to examine the effect on 3Rs behaviour. The findings in this research presented that habit contributed the most to the variation of 3Rs behaviour because its beta value was the highest at 0.354 as compared to other variables. Therefore, this study's findings can add on to the past studies and provide reference to the environmental issues with this theoretical model.

This study revealed that the R-square value was 0.571 for 3Rs behavioural intention and 0.425 for 3Rs behaviour. It indicated that 57.1% and 42.5% of the total variation in the dependent variables (behavioural intention and behaviour) can be described by the independent variables (attitude, subjective norm, perceived behavioural control, facilitating conditions, habit, and behavioural intention). The R^2^s were even more significant as compared to the prior research conducted by Strydom (2018) with the R^2^s of 46.4% and 26.4% only. This can further justify that the model of this study was fit and appropriate.

### 5.8 Practical implications

Among the variables, attitude is found to be the best predictor towards 3Rs behavioural intention.

As the items for the construct attitude were more related to the affective component of the attitude, it is important to build the environmental responsibility feeling among the Malaysian citizens. Hence, government can educate and embed society's mind-set of 3Rs behaviour in plastic usage of Malaysians through social media such as Facebook, Instagram, Twitter, etc. Awareness also can be achieved by providing sufficient information related to 3Rs concept through television, radio, newspapers, and schools. Besides that, the attitude of the manufacturers is also very important. Manufacturers with a good attitude toward 3Rs behavioural intention are more motivated to reduce the usage of material and to select more recyclable materials. According to Friedrich (2020), one way to reduce the environmental impact from plastic packaging is to replace plastic with biobased substitute materials. Malaysian Government also can consider the implementation of extended producer responsibility (EPR), a strategy that makes manufacturers assume the responsibility for the cost related to disposal of the waste (Kunz et al., 2018). According to Diggle and Walker (2020), the Canadian federal government is dedicated to draw near to zero plastic waste through the implementation of EPR across the country to reduce single-use plastics (SUPs) food wrapping pollution.

Although facilitating conditions was the least significant among the variables towards the behaviour, it still portrayed a significant relationship. According to Triandis (1977), facilitating conditions play a significant role in achieving behaviour of the 3Rs in plastic usage. Referring to Table 2, PBC 6 ‘I know the place to take my plastic for recycling’ had second lowest mean in perceived behavioural control variable. It indicated that many of the respondents did not know the place to send plastic for recycling. Thus, it is suggested that government or NGOs should improve and increase the number of recycling facilities in residential areas such as recycling bin. It could encourage Malaysians to apply the 3Rs in plastic usage.

Habit is found to be the best predictor variable of 3Rs behaviour in this research. Therefore, government and corporations are recommended to cultivate Malaysians to be habitual on 3Rs behaviour in plastic usage. Ministry of Education Malaysia (MOE) should embed the 3Rs concept as mandatory education materials, so that Malaysians can be cultivated with 3Rs concept from their childhood. Policy should be tightened on the usage of single-used plastic. For example, to reduce plastic usage, free plastic bags should be forbidden in all supermarkets and retail shops throughout Malaysia and for all days of the week. Levy for the plastic bags should be increased from the existing rate of 20 cents to at least 50 cents, so that consumers are reminded to bring recycling bags when shopping and make it a habit to do so. Also, disposable Styrofoam lunch boxes and cups should be banned and replaced with a recyclable plastic container which needs to be purchased when buying food. This is to encourage consumers to bring their food containers and tumblers to pack food and drinks and build a habit. The Government could also impose a higher tax charge on plastic products that are not recyclable. Thus, consumers are made to pay for a higher price for plasticware and encouraged to buy better quality plasticware and reuse them instead of keeping buying new ones. This could also help to reduce plastic waste. The Government should also make the separation of recyclable waste for every household and commercial lots mandatory in order to foster a recycling habit to all Malaysians. Thus, recycling facilities such as recycling bins should be prepared by the authorities and properly managed. All these measures should be monitored closely and fine should be imposed on non-compliance.

This research can contribute to Malaysia Government to have better and effective strategies or policies to deal with the environmental pollution caused by plastic waste. It helps to build environmentally responsible Malaysian citizens which can lead to a better living environment for future generation.

### 5.9 Limitations of the study

This research has a few limitations. Firstly, this research applied cross-sectional approach, which is used to figure out what is happening in the present time and usually collect data once (Rindfleisch et al., 2008). Since the data is only collected once, the findings may not be used to understand the cause-and-effect factors and to generalise for future periods. Self-administered questionnaire method with closed-ended questions was applied. It restrained constructive feedbacks from the respondents as the questionnaire was designed in the pre-set rating model. It caused limitation on information and opinions to be provided by the respondents. Therefore, respondents might not respond with a precise opinion relating to the issue in this study. Thus, the usefulness of this study might be limited.

### 5.10 Recommendation

Future researchers may collect data using a longitudinal approach instead of cross-sectional approach. This collection process is more accurate since data is collected in real-time using observational data. Longitudinal approach helps researchers find out behaviour patterns that may occur over long periods of time. These studies have high levels of data validity. Due to the time and resource constraint, we were unable to conduct a longitudinal study. Referring to Mathers et al. (2007), it will consume few months or a year in data collection for longitudinal approach.

According to Malaysia Department of Statistic, there are 32.4 million citizens in Malaysia. Noncitizens contribute 10.38% (3.4 million) of the total population. As those non-citizens reside in Malaysia, they will also contribute to the waste generated in Malaysia. Therefore, future researchers may include non-citizens in their future researches.

In this research, close-ended questionnaire was used, but it is suggested to practice open-ended questionnaire by the future researchers. An open-ended questionnaire is able to collect more meaningful answers from the respondents, which allows them to convey their knowledge and feelings as opposed to closed-ended questionnaire which only allows predetermined answers. The research can also be done in a more specific industry. There are certain sectors which consume large amounts of plastic, and therefore, future researches can be done in a more specific industry such as hotels, restaurants, hospital, educational institutions, industries, etc. Organisations and intermediaries also could be studied for the implication of the 3Rs concept on plastic usage. Besides, current research only focused on the 3Rs concept on plastic usage while the future researches can discover in other recycling materials like glass, paper, and aluminium.

## 6 Conclusion

In a nutshell, the result in this study shows that there is a significant and positive relationship between attitude, subjective norm, perceived behavioural control, 3Rs behavioural intention, habit, facilitating conditions, and 3Rs behaviour. Thus, the specific and general objectives were achieved. It is important to provide sufficient information related to 3Rs concept through social media, television, radio, newspapers, and schools. In order to encourage Malaysians to apply the 3Rs concept in plastic usage, the number of recycling facilities in residential areas such as recycling bins should be increased. Government also needs to implement better strategies and effective policies to deal with the environmental pollution caused by plastic waste. Supporting laws and regulations are required to encourage recycling activities as well as changing Malaysians’ behaviour.

## Declarations

### Author contribution statement

Krishna Moorthy: Conceived and designed the experiments; Contributed reagents, materials, analysis tools or data.

Wong Zhi Ying, Chin Wei Khong, Gan Zhao Chern, Thong Zin Lin: Performed the experiments; Analyzed and interpreted the data.

Foo Pik Yin: Analyzed and interpreted the data.

Chin Yoon Mei: Analyzed and interpreted the data; Wrote the paper.

### Funding statement

This research did not receive any specific grant from funding agencies in the public, commercial, or not-for-profit sectors.

### Data availability statement

Data will be made available on request.

### Declaration of interests statement

The authors declare no conflict of interest.

### Additional information

No additional information is available for this paper.
