# Supplementary material for: Determinants of 3Rs behaviour in plastic usage: A study among Malaysians
Source: Heliyon. 2020 Dec 23;6(12):e05805. doi: 10.1016/j.heliyon.2020.e05805 (PMC7773869; doi:10.1016/j.heliyon.2020.e05805)
Supplement: Supplementary file 1 — APPENDIX [file mmc1.docx]

**APPENDIX**

**Survey Questions**

| H1 | Attitude |
| --- | --- |
| AT1 | Applying the 3Rs concept on plastic usage is good. |
| AT2 | Applying the 3Rs concept on plastic usage is useful. |
| AT3 | Applying the 3Rs concept on plastic usage is responsible. |
| AT4 | I like the idea of 3Rs (reduce, reuse, recycle). |
| AT5 | I think applying the 3Rs concept on plastic usage has many positive effects on the environment. |
| H2 | Subjective Norm |
| SN1 | Most people who are important to me think that I should apply the 3Rs concept on a daily basis. |
| SN2 | Most of my family members think that 3Rs concept is a good thing to follow. |
| SN3 | Most of my friends and acquaintances think that 3Rs concept is a good thing to follow. |
| SN4 | The culture in this country encourages application of the 3Rs concept on plastic usage. |
| H3 | Perceived Behavioural Control |
| PBC1 | The application of the 3Rs concept on plastic usage is under my control. |
| PBC2 | I am willing to apply the 3Rs concept on plastic usage. |
| PBC3 | I think I need to apply the 3Rs concept on plastic usage at home. |
| PBC4 | I should not waste anything if it could be used again. |
| PBC5 | I know what item can be reduced, reused and recycled. |
| PBC6 | I know the place to take my plastic for recycling. |
| H4 | Behaviour Intention |
| BI1 | I am willing to reduce the use of plastic. |
| BI2 | I am planning to recycle plastic products. |
| BI3 | I will try to reuse plastic, if possible. |
| BI4 | I am willing to participate in the 3Rs programme. |
| BI5 | I will make an effort to apply the 3Rs concept on plastic usage. |
| H5 | Habit |
| HB1 | Applying the 3Rs concept on plastic usage is something I do automatically. |
| HB2 | Applying the 3Rs concept on plastic usage is something I do without thinking. |
| HB3 | I am applying the 3Rs concept on plastic usage without consciously remember. |
| HB4 | I start applying the 3Rs concept on plastic usage before I realise I am doing it. |
| H6 | Facilitating Conditions |
| FC1 | I will apply the 3Rs concept on plastic usage, if time permits. |
| FC2 | I will apply the 3Rs on plastic usage, if there is space in my house. |
| FC3 | I will apply the 3Rs concept on plastic usage, if recycling facilities are provided. |
| FC4 | I will apply the 3Rs concept on plastic usage, if I can save money. |
| DV | Behaviour |
| BI1 | I will try to bring reusable bags when shopping. |
| BI2 | I will purchase unpackaged fruits and vegetables or those with very little packaging. |
| BI3 | I always reuse old containers. |
| BI4 | I will try to repair an item instead of buying new items. |
| BI5 | I always reuse the single use plastic bag for garbage collection. |
| BI6 | I seldom buy disposable products. |
